# Magnesium supplementation did not reduce serum calciprotein crystallization and arterial stiffness in individuals with type 2 diabetes: a randomized, double-blind, placebo-controlled trial

**DOI:** 10.1016/j.ajcnut.2026.101299

**Published:** 2026-03-26

**Authors:** Romain Meer, Joyce Y Xu, Simon P Newsom, Andreas Pasch, Marc G Vervloet, Pim A de Jong, Petra JM Elders, Joline WJ Beulens

**Affiliations:** 1 Department of Epidemiology & Data Science, Amsterdam UMC, Amsterdam, The Netherlands; 2 Amsterdam Cardiovascular Sciences Research Institute, Amsterdam, The Netherlands; 3 Department of Nephrology, Amsterdam UMC, Amsterdam, The Netherlands; 4 Laboratory of Specialized Diagnostics & Research, Department of Laboratory Medicine, Amsterdam UMC, Amsterdam, The Netherlands; 5 Calciscon AG, Biel, Switzerland; 6 Institute for Physiology and Pathophysiology, Johannes Kepler University Linz, Linz, Austria; 7 Department of Nephrology, Radboud University Medical Centre, Nijmegen, The Netherlands; 8 Department of Radiology, University Medical Centre Utrecht, Utrecht, The Netherlands; 9 Department of General Practice, Amsterdam UMC, Amsterdam, The Netherlands; 10 Amsterdam Public Health Research Institute, Amsterdam, The Netherlands; 11 Julius Centre for Health Sciences and Primary Care, University Medical Centre Utrecht, Utrecht, The Netherlands

**Keywords:** randomized controlled trial, magnesium citrate, calciprotein crystallization, arterial stiffness, arterial calcification, carotid-femoral pulse wave velocity, T_50_, diabetes mellitus type 2

## Abstract

**Background:**

Medial arterial calcification (MAC) and increased arterial stiffness contribute to cardiovascular disease risk in type 2 diabetes mellitus (T2DM). Experimental studies suggest that magnesium supplementation may halt arterial calcification and improve arterial stiffness.

**Objectives:**

This study aimed to evaluate the effect of 6-mo oral magnesium citrate supplementation on calciprotein crystallization (T_50_) and carotid-femoral pulse wave velocity (cfPWV) in individuals with T2DM and peripheral MAC.

**Methods:**

This double-blind, placebo-controlled trial randomly assigned 74 participants with T2DM [78% males, 72 (68–76) y] with peripheral MAC and cfPWV≥12.0 m/s to magnesium citrate (350 mg/d; *n* = 37) or placebo (*n* = 37). Nephelometry-based T_50_ measurements, cfPWV measurements, and 24-h urine collections were obtained at baseline, 3 and 6 mo. Longitudinal analysis of covariance adjusted for baseline T_50_ and cfPWV was used to study the treatment effects on T_50_ and cfPWV.

**Results:**

Baseline mean T_50_ and cfPWV were similar between the magnesium group (T_50_ 348 ± 54 min; cfPWV 15.9 ± 2.2 m/s) and the placebo group (362 ± 54 min; 15.6 ± 2.0 m/s). Magnesium in serum and in 24-h urine were lower in the magnesium group [0.74 (0.71–0.77) mmol/L and 3.30 (2.06–4.71) mmol/24 h] compared with the placebo group [0.81 (0.74–0.86) mmol/L and 4.31 (3.09–5.54) mmol/24 h]. Supplementation increased 24-h urine magnesium excretion (*P* < 0.001), but not serum magnesium concentration (*P* = 0.073) over time in the magnesium group relative to the placebo group. Magnesium supplementation did not increase T_50_ [ẞ = 6 min (–11, 22), *P* = 0.491] but did increase cfPWV [ẞ = 0.8 m/s (0.1, 1.5), *P* = 0.021] over 6 mo in the magnesium group relatively to the placebo group, but the statistical significance was lost after adjusting for clinically relevant baseline differences [T_50_: ẞ = 7 min (–12, 25), *P* = 0.482; cfPWV: ẞ = 0.5 m/s (–0.2, 1.3), *P* = 0.180].

**Conclusions:**

Six-month magnesium citrate supplementation did not reduce calciprotein crystallization and arterial stiffness in older individuals with T2DM with peripheral MAC. Daily supplementation of 350 mg appears to be ineffective in this population, possibly attributable to normomagnesemia and preserved renal function.

This study was registered at the Dutch Trial Register (CCMO) as NL81281.029.22 and at ISRCTN as 60460377.

## Introduction

Cardiovascular disease (CVD) is the main cause of mortality in individuals with type 2 diabetes mellitus (T2DM) [1]. This may be attributed to a high arterial calcification burden, which confers a 3- to 4-fold increased CVD risk [2,3].

Individuals with T2DM develop medial arterial calcification (MAC), a distinct form of calcification caused by mineralization of the elastin fibers in the tunica media [4]. MAC differs from atherosclerotic intimal arterial calcification (IAC) in anatomic location, pathogenesis, and clinical consequences [4]. MAC’s multifactorial pathogenesis involves a disbalance of phosphate and calcium, partly attributable to renal dysfunction [5]. MAC contributes to arterial stiffening [6], white matter lesions [7], lower-limb amputation [8], chronic kidney disease (CKD) [4], and foot ulcers [4,8]. Magnesium interferes with mineral metabolism involved in MAC, and supplementation could therefore be promising and warrants further physiological and experimental elaboration.

Physiological evidence shows that magnesium prevents the loss of calcification inhibitors [9,10], inhibits osteogenic transcription factor expression [9,10], inhibits hydroxyapatite formation from calcium and phosphate [[11], [12], [13], [14]], and inhibits primary to secondary calciprotein particle (CPP) transformation [15,16]. These inhibitory effects are relevant as they prevent vascular smooth muscle cell differentiation into an osteogenic phenotype, a key process which makes MAC pathophysiologically distinct from IAC [4,17]. Although magnesium shows promise as a safe and affordable treatment to reduce MAC, randomized clinical trials (RCTs) assessing its impact on coronary artery calcification (CAC) progression have yielded conflicting results [[18], [19], [20]]. Moreover, a meta-analysis showed no significant treatment effects of magnesium supplementation on carotid-femoral pulse wave velocity (cfPWV), a measure of central arterial stiffness [[21], [22], [23]]. Nevertheless, subgroup analyses revealed promising results in individuals with comorbidities common in T2DM, such as hypertension, CKD, and coronary artery disease. Magnesium might therefore have a beneficial effect on arterial calcification and arterial stiffness in individuals with T2DM.

Recently, calciprotein crystallization time (T_50_) has been proposed as a novel biomarker for calcification propensity [24]. This assay is relatively low-cost and radiation-free, rendering it more suitable for routine clinical practice and large-scale epidemiological studies compared with advanced imaging methods [25]. The T_50_, representing the one-half maximum transition time for primary to secondary CPPs, is expressed in minutes, with a lower T_50_ indicating faster calciprotein crystallization [25,26]. The T_50_ may also be used as a surrogate endpoint for arterial calcification, as it is associated with aortic calcification mass score, Agatston score [27], CAC score [28], and cardiovascular and all-cause mortality [29]. A previous RCT using vitamin K was unsuccessful in improving T_50_ [30], whereas trials with phosphate binders [[31], [32], [33], [34]], and dietary modifications [35,36], showed improvement of T_50_. Moreover, an RCT using magnesium supplementation showed improvement of T_50_ in patients with CKD, though without assessing arterial stiffness [37]. We hypothesize that magnesium can increase T_50_ and lower arterial stiffness in T2DM. Therefore, the objective of this trial was to evaluate the treatment effect of 6-mo oral magnesium supplementation on *1*) T_50_ and *2*) arterial stiffness in individuals with T2DM, MAC, and stiff arteries.

## Methods

### Study design

In this randomized, double-blind, placebo-controlled clinical trial, participants were recruited from the Diabetes Care System (DCS) cohort. The DCS, described elsewhere [38], monitors glycemic control, diabetes-related risk factors, and complications annually (1998–2019) and triennially (2020–2022). DCS participants previously consenting to be contacted for future research were invited to the Early-HFpEF study (2019–2023; METC VUmc, 2018.064—EARLY-HFpEF) [6], in which peripheral arterial calcification data via computed tomography (CT) were collected. Early-HFpEF participants were invited to participate in the Mg-MAC trial (2023–2024). The Mg-MAC trial was conducted in agreement with the principles of the Declaration of Helsinki and was in accordance with the Medical Research Involving Human Subjects Act. The study was approved by the Medical Ethical Committee VUmc and is registered in the Dutch Trial Register (CCMO, NL81281.029.22). Evaluations occurred at baseline, 3 mo (follow-up 1), and 6 mo (follow-up 2). All participants provided written informed consent.

### Study population

Participants were eligible when they were between 50 and 80 y old and had MAC in either the femoral and/or crural arteries. The presence of MAC and the quantity of lower-extremity arterial calcification were assessed in the Early-HFpEF study using a CT-guided histologically validated scoring algorithm [39]. Details about these calcification measurements can be found elsewhere [6]. Exclusion criteria included severe diabetes complications (e.g., atrial fibrillation, neuropathy, nephropathy, and retinopathy), significant comorbidities (e.g., heart failure, hepatic insufficiency, myasthenia gravis, previous aortic surgery, inflammatory bowel disease, chronic diarrhea, hyperthyroidism, and active parathyroid disease), and life expectancy <3 y, use of medication interfering with magnesium (e.g., levothyroxine, tiludronate, alendronate, and warfarin) and unwillingness to stop magnesium supplementation (e.g., antacids). A washout period of minimally 14 d was implemented between screening and baseline for individuals who reported the use of magnesium supplementation during screening (*n* = 15). Screening included confirmation of cfPWV ≥12.0 m/s and serum magnesium levels within the 0.62–1.07 mmol/L range.

### Intervention

Participants were randomly assigned 1:1 by an independent statistician to either oral magnesium citrate [3 × 117 mg capsules daily (equal to 14.4 mmol magnesium); Medisan, Heerenveen, The Netherlands] or placebo for 6 mo. Stratified block randomization was done by stratification on sex, age (≤72 compared with >72 y), and cfPWV (<14.0 compared with ≥14.0 m/s), creating 8 strata of approximately equal size, whereas block randomization with block size 4 allowed numerical balance in treatment assignment. Placebo capsules matched the active treatment in taste and appearance. Compliance, calculated as (*pills prescribed – pill count) / pills prescribed × 100*), was assessed via returned capsules, and participants with <80% compliance were deemed noncompliant.

### Calciprotein crystallization time test (T_50_)

The primary outcome was the difference in change of T_50_ from baseline to month 6 between magnesium and placebo. T_50_, measured at baseline and both follow-up visits, was determined via nephelometry on serum samples stored at –80°C and shipped post-trial, as described by Pasch et al. [24]. Frozen samples stored at –80°C are suitable for this assay and research purposes. The coefficient of variation was 8.6% [24,40]. High agreement was observed between the Amsterdam UMC and the original laboratory (Calciscon AG) with a Passing Bablok regression slope of 1.002 (0.948–1.059) based on 21 samples [[40], [41]].

### Arterial stiffness

The secondary outcome was the difference in change of cfPWV from baseline to month 6 between magnesium and placebo. The following vascular parameters were measured: brachial systolic and diastolic blood pressure (SBP, DBP), augmentation index (AIX), heart rate-corrected augmentation index (AIX75), and cfPWV. SBP and DBP were measured in the supine position in triplicate using an automated SphygmoCor XCEL oscillomat (AtCor) with 30-s intervals. Subsequently, the AIX and AIX75 were measured. cfPWV was measured in duplicate, with a third measurement if consecutive measurements differed by >1.0 m/s. Mean values were computed for analysis. According to the ARTERY Society guidelines [42] and the ARTERY PWV validation guidelines [43], femoral cuff-based cfPWV measurements were validated compared with applanation tonometry-based cfPWV measurements, demonstrating high correlation (*r* = 0.93) and excellent accuracy (mean difference <0.5 m/s, SD of difference <0.8 m/s).

### Biochemical measurements

Fasting blood samples were drawn at each visit and analyzed at DiagnostIQ. At baseline and month 6, measurement of potassium, sodium, urea, calcium, phosphate, total cholesterol, triglycerides, HDL cholesterol, LDL cholesterol, magnesium, glucose (all mmol/L), creatinine (μmol/L), creatinine clearance (mL/min), albumin, total protein (both g/L), and glycated hemoglobin (HbA1c) (mmol/mol) were measured using Capillarys (HbA1c) and Cobas 8000. At month 3, only serum magnesium was measured. The estimated glomerular filtration rate (eGFR) (mL/min per 1.73 m^2^) was estimated using the 2009 Chronic Kidney Disease Epidemiology Collaboration (CKD-EPI) formula [44].

In addition, 24-h urine samples, collected the day before baseline and 3- and 6-mo visits, were analyzed at DiagnostIQ for volume (mL), creatinine excretion (mmol/24 h), and level (mmol/L), and magnesium excretion (mmol/24 h) using Cobas 8000. Details on the biochemical analyses can be found elsewhere [45].

### Covariates

Diabetes duration (y) and smoking status (ever/never) were obtained via linkage to DCS routine care data closest to the screening visit. Alcohol consumption (yes/no) and diet quality were measured via the Dutch Healthy Diet Index 2015 food frequency questionnaire (DHD-15 FFQ) administered at baseline and at month 6. This validated FFQ captures adherence to the Dutch dietary guidelines of 2015 into a score of 0 (no adherence) to 150 (total adherence). This FFQ was validated compared with 3-d food records with acceptable agreement [46]. Moreover, the history of cerebrovascular disease (yes/no), history of coronary artery disease (yes/no), femoral and crural arterial calcification pattern (MAC/non-MAC), and peripheral calcification score (Agatston units) was obtained from the Early-HFpEF study. Detailed information on these covariates, especially the calcification parameters, can be found in previous published work [6]. CT-based arterial calcification outcomes were not considered during the trial, as this study focused on the endpoints of arterial calcification (T_50_) and arterial stiffness (cfPWV).

### Statistical analysis

Baseline characteristics were described for both intervention arms. Continuous variables were reported depending on their distribution using mean ± SD for normally distributed variables or median (IQR) for nonnormally distributed variables. Categorical variables were presented as frequency (percentage). Significant differences between intervention arms were not tested as suggested by the CONSORT 2010 statement [47]. However, baseline characteristics were inspected for clinically relevant differences across treatment arms.

As primary analysis, the effect of magnesium citrate compared with placebo on T_50_ was evaluated using a linear mixed model, specifically a longitudinal analysis of covariance with adjustment for baseline T_50_ and with an intention-to-treat approach (analyzing all participants conforming to their group allocation, regardless of their compliance). Three different effect estimates were determined: the overall intervention effect and the intervention effect over both follow-up periods. The 95% confidence intervals and *P* values over both follow-up periods were obtained with different reference categories for time: the baseline study visit as reference for the overall treatment effect and treatment effect over the first follow-up period, and the follow-up 1 study visit as reference for the treatment effect over the second follow-up period. Standard assumptions of linear mixed model, such as linearity, normality of residuals, homoscedasticity, and multicollinearity, were checked with standard tests (partial plots, histograms of residuals, QQ-plots, and variance inflation factors).

As secondary analysis, the effect of magnesium citrate compared with placebo on cfPWV was evaluated with a similar methodology as the primary analysis. Moreover, additional adjustment for time-varying SBP was performed to evaluate the treatment effect independently of blood pressure. As tertiary analysis, the treatment effect on serum mineral levels, renal function markers, lipid regulation markers, and glucose regulation markers was estimated, however only by evaluation of the overall intervention effect. Two sensitivity analyses were conducted: a per-protocol analysis excluding participants with <80% compliance, and an analysis adjusting for clinically relevant between-group differences at baseline.

Effect modification by sex and history of CVD was evaluated by constructing the interaction term with the treatment group. In case of *P* ≤ 0.10, the stratified effect estimates were reported [48].

No imputation of missing data was performed as longitudinal analysis of covariance can appropriately deal with missing data by conducting available-case analysis, assuming that data are missing at random and the model is correctly specified [48]. All analyses were carried out using R software (Version 4.3., R) and blinded to treatment allocation. Log transformation was applied where necessary and based on normality of the outcome variable and the residuals of the regression, as assessed using histograms. Statistical significance was defined as *P* ≤ 0.05.

### Sample size calculation

The sample size was calculated based on studies on T_50_ [37], and cfPWV [23], and performed by an independent statistician (Department of Epidemiology & Data Science, Amsterdam UMC). On the basis of an SD of change in T_50_ of 50 min and in cfPWV of 1.16 m/s, 29 participants per arm as randomized 1:1 were sufficient to detect a 15% change in T_50,_ and 21 participants per arm were sufficient to detect a 11% change in cfPWV with an *α* of 0.05 and 80% power. The target sample size was 58 participants, but 74 were recruited to account for a 20% dropout rate and to ensure sufficient power for both outcomes.

## Results

### Baseline characteristics

**FIGURE 1:**
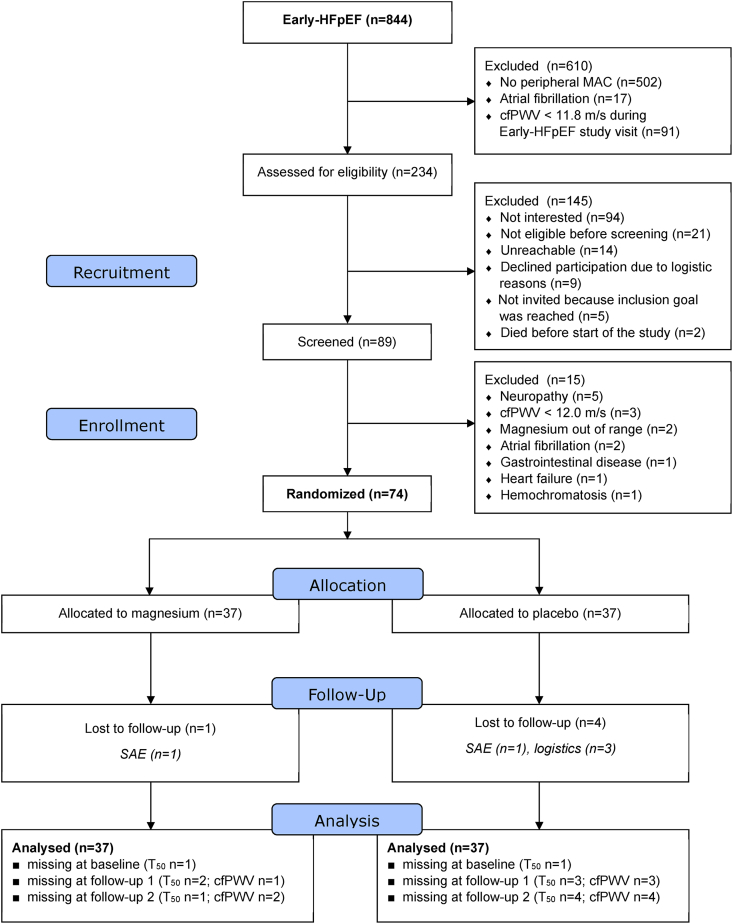
CONSORT 2010 flow diagram of study participation. cfPWV, carotid-femoral pulse wave velocity; MAC, medial arterial calcification; T_50_, calciprotein crystallization time; SAE, serious adverse event.

A total of 234 individuals were invited, of which 89 were screened, and 74 participants were enrolled (Figure 1). Sixty-nine participants completed the study, with one participant missing the first follow-up visit. Five participants were lost to follow-up related to logistics and to serious adverse events (Figure 1), and this was not different across treatment arms (*P* = 0.358). Six participants (magnesium *n* = 4; placebo *n* = 2) were considered noncompliant.

The study included 58 (78%) male participants with a median age of 72 (68–76) y and a median T2DM duration of 18 (14–21) y (Table 1). Baseline characteristics were similar across treatment arms. Overall diet quality was similar at baseline and did not change over the course of 6 mo. Clinically relevant differences across treatment arms were observed for eGFR, serum magnesium levels (both lower in the magnesium group), HbA1c levels, and total peripheral calcification (both higher in the magnesium group). Mean T_50_ and mean cfPWV were similar between the magnesium (T_50_ 348 ± 54 min; cfPWV 15.9 ± 2.2 m/s) and placebo group (T_50_ 362 ± 54 min; cfPWV 15.6 ± 2.0 m/s).

**TABLE 1:** Baseline characteristics for the 74 individuals who were randomly assigned to either magnesium citrate (*n* = 37) arm or placebo (*n* = 37)

Baseline characteristics	Magnesium (*n* = 37)	Placebo (*n* = 37)
Demographic variables		
Sex, male (yes)	31 (83.8%)	27 (73.0%)
Age (y)	72 (69–76)	72 (68–75)
Duration of diabetes (y)	18 (15–22)	18 (14–21)
Height (cm)	177 ± 8	175 ± 10
Manifest CVD		
Cerebrovascular disease (yes)^1^	0 (0.0%)	3 (9.7%)
Coronary artery disease (yes)^1^	6 (17.6%)	5 (16.1%)
Diet		
DHD-15 Score (unitless)^1^	99 ± 17	101 ±18
Alcohol consumer (yes)^1^	23 (63.9%)	21 (56.8%)
Ever smoker (yes)^1^	28 (77.8%)	24 (64.9%)
Laboratory measurements		
Potassium (mmol/L)^1^	4.4 (4.1–4.6)	4.2 (4.0–4.4)
Sodium (mmol/L)^1^	139 (138–140)	139 (138–140)
Urea (mmol/L)^1^	5.9 (4.9–7.8)	5.4 (4.8–6.1)
Creatinine (μmol/L)^1^	84 (71–105)	74 (68–88)
Creatinine clearance (mL/min)^1^	97 (78–124)	111 (92–136)
eGFR (mL/min/1.73 m^2^)^1^	82 (67–92)	90 (77–96)
Calcium (mmol/L)^1^	2.29 (2.23–2.36)	2.26 (2.21–2.33)
Phosphate (mmol/L)^1^	1.06 (0.95–1.14)	1.03 (0.97–1.10)
Total cholesterol (mmol/L)^1^	3.8 (3.3–5.0)	4.0 (3.3–4.8)
Triglycerides (mmol/L)^1^	1.5 (1.1–1.8)	1.4 (0.9–1.8)
HDL cholesterol (mmol/L)^1^	1.1 (1.0–1.4)	1.2 (1.0–1.7)
LDL cholesterol (mmol/L)^1^	2.0 (1.6–2.8)	2.0 (1.6–2.4)
Albumin (g/L)^1^	45 (44–46)	44 (43–46)
Total protein (g/L)^1^	71.7 (69.6–74.6)	71.0 (67.8–72.4)
Magnesium (mmol/L)^1^	0.74 (0.71–0.77)	0.81 (0.74–0.86)
HbA1c (mmol/mol)^1^	57 (51–65)	52 (47–59)
Fasting glucose (mmol/L)^1^	9.0 (7.9–10.2)	8.2 (7.0–10.0)
Vascular measurements		
Systolic blood pressure (mmHg)	152 ± 15	150 ± 17
Diastolic blood pressure (mmHg)	79 ± 8	82 ± 9
Augmentation Index (unitless)	28 ± 8	30 ± 8
Augmentation Index 75 (unitless)	23 ± 10	25 ± 9
cfPWV (m/s)	15.9 ± 2.2	15.6 ± 2.0
Calcification parameters		
T_50_ (min)^1^	348 ± 54	362 ± 54
Presence of femoral MAC (yes)	32 (86.5%)	33 (89.2%)
Presence of crural MAC (yes)^1^	31 (83.8%)	23 (63.9%)
Total peripheral calcification (Agatston units)^1^	5029 (2301–11,763)	2770 (762–7111)
24-h urine parameters		
Total volume (L)^1^	1.8 (1.4–2.3)	2.2 (1.7–2.8)
Creatinine per 24 h (mmol/24 h)^1^	12.5 (10.9–14.9)	12.5 (10.5–14.4)
Creatinine in 24 h urine (mmol/L)^1^	7.2 (5.0–9.5)	5.7 (4.6–6.6)
Magnesium per 24 h (mmol/24 h)^1^	3.3 (2.1–4.7)	4.3 (3.1–5.5)

Continuous variables were reported as mean ± SD or median (IQR), depending on their distribution. Categorical variables were presented as frequency (group percentage).

Abbreviations: cfPWV, carotid-femoral pulse wave velocity; CVD, cardiovascular disease; DHD-15 Score, Dutch Healthy Diet Index Score; eGFR, estimated glomerular filtration rate; HbA1c, glycated hemoglobin; MAC, medial arterial calcification; T_50_, calciprotein crystallization time.

1 Missings: cerebrovascular disease (*n* = 9), coronary artery disease (*n* = 9), DHD-15 Score (*n* = 1), alcohol consumer (*n* = 1), ever smoker (*n* = 1), potassium (*n* = 2), sodium (*n* = 2), urea (*n* = 2), creatinine (*n* = 2), creatinine clearance (*n* = 2), eGFR (*n* = 2), calcium (*n* = 2), phosphate (*n* = 2), total cholesterol (*n* = 2), triglycerides (*n* = 2), HDL cholesterol (*n* = 2), LDL cholesterol (*n* = 2), albumin (*n* = 2), total protein (*n* = 2), magnesium (*n* = 2), HbA1c (*n* = 2), fasting glucose (*n* = 2), T_50_ (*n* = 2), presence of crural MAC (*n* = 1), total peripheral calcification (*n* = 1), total volume urine (*n* = 1), creatinine per 24 h urine (*n* = 1), creatinine clearance 24 h urine (*n* = 1), magnesium per 24 h urine (*n* = 1).

### Magnesium status

**FIGURE 2:**
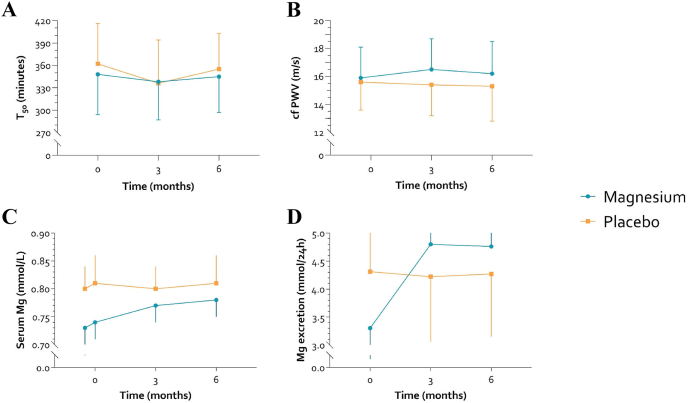
Longitudinal data of the arterial calcification biomarker that is T_50_ (A), the arterial stiffness parameter that is cfPWV (B), serum Mg levels and (C) 24-h urine Mg excretion (D) stratified per treatment arm: magnesium (blue) and placebo (orange). cfPWV, carotid-femoral pulse wave velocity; Mg, magnesium; T_50_, calciprotein crystallization time.

Magnesium excretion in 24-h urine increased from 3.30 (2.06–4.71) mmol/L to 4.80 (3.97–7.04) at follow-up 1 and to 4.76 (3.40–6.98) at follow-up 2 in the magnesium group (Figure 2 and Table 2). In contrast, the urinary magnesium excretion remained stable around 4.30 mmol/L in the placebo group. Accounting for baseline differences between treatment arms, the difference in increase was statistically significant (*P* < 0.001).

**TABLE 2:** Results of the longitudinal analysis of covariance for biochemical parameters and cfPWV

Longitudinal analysis of covariance (1/2)	Magnesium (*n* = 37)	Placebo (*n* = 37)	Unadjusted *β* (95% CI), *P* value	Adjusted *β* (95% CI), *P* value
T_50_ (min)				
Baseline	348 ± 54	362 ± 54	6 (–11, 22)	7 (–12, 25)
Follow-up 1	338 ± 51	336 ± 58	0.491	0.482
Follow-up 2	345 ± 48	355 ± 48		
cfPWV (m/s)				
Screening	15.9 ± 2.2	15.6 ± 2.0	0.8 (0.1, 1.5)	0.5 (–0.2, 1.3)
Follow-up 1	16.5 ± 2.2	15.4 ± 2.2	0.021	0.180
Follow-up 2	16.2 ± 2.3	15.3 ± 2.5		
Magnesium serum (mmol/L)				
Screening	0.73 (0.68–0.77)	0.80 (0.76–0.84)	0.01 (–0.02, 0.04)	–0.03 (–0.07, 0.00)
Baseline	0.74 (0.71–0.77)	0.81 (0.74–0.86)	0.436	0.073
Follow-up 1	0.77 (0.74–0.81)	0.80 (0.78–0.84)		
Follow-up 2	0.78 (0.75–0.81)	0.81 (0.77–0.86)		
Potassium (mmol/L)				
Baseline	4.4 (4.1–4.6)	4.2 (4.0–4.4)	0.2 (0.0, 0.3)	0.1 (0.0, 0.2)
Follow-up 2	4.5 (4.3–4.6)	4.3 (4.1–4.4)	0.020	0.172
Sodium (mmol/L)				
Baseline	139 (138–140)	139 (138–140)	0 (–1, 1)	1 (0, 2)
Follow-up 2	139 (137–140)	139 (138–140)	0.762	0.254
Urea (mmol/L)				
Baseline	5.9 (4.9–7.8)	5.4 (4.8–6.1)	1.1 (1.0, 1.2)^1^	1.1 (0.9, 1.2)^1^
Follow-up 2	6.6 (5.4–8.0)	5.4 (4.6–6.6)	0.225	0.286
Creatinine (μmol/L)				
Baseline	84 (71–105)	74 (68–88)	1 (–4, 6)	2 (–4, 7)
Follow-up 2	89 (71–110)	73 (62–89)	0.644	0.525
Creatinine clearance (mL/min)				
Baseline	97 (78–124)	111 (92–136)	–5 (–19, 8)	–8 (–23, 6)
Follow-up 2	93 (73–116)	103 (88–127)	0.433	0.259
eGFR (ml/min/1.73 m^2^)				
Baseline	82 (67–92)	90 (77–96)	–2 (–5, 1)	–2 (–6, 1)
Follow-up 2	77 (58–93)	89 (75–97)	0.211	0.182
Calcium (mmol/L)				
Baseline	2.29 (2.23–2.36)	2.26 (2.21–2.33)	0.02 (–0.02, 0.06)	0.02 (–0.03, 0.06)
Follow-up 2	2.32 (2.26–2.37)	2.28 (2.24–2.33)	0.313	0.439

Longitudinal analysis of covariance (2/2)	Magnesium (*n* = 37)	Placebo (*n* = 37)	Unadjusted *β* (95% CI), *P* value	Adjusted *β* (95% CI), *P* value

Phosphate (mmol/L)				
Baseline	1.06 (0.95–1.14)	1.03 (0.97–1.10)	–0.04 (–0.10, 0.02)	–0.02 (–0.09, 0.05)
Follow-up 2	1.02 (0.97–1.10)	1.06 (0.95–1.11)	0.235	0.504
Total cholesterol (mmol/L)				
Baseline	3.8 (3.3–5.0)	4.0 (3.3–4.8)	0.0 (–0.2, 0.2)	–0.1 (–0.3, 0.2)
Follow-up 2	4.0 (3.4–4.9)	4.0 (3.6–4.5)	0.736	0.623
Triglycerides (mmol/L)				
Baseline	1.5 (1.1–1.8)	1.4 (0.9–1.8)	0.0 (–0.3, 0.2)	–0.1 (–0.4, 0.2)
Follow-up 2	1.4 (0.9–1.8)	1.4 (0.9–1.7)	0.767	0.373
HDL cholesterol (mmol/L)				
Baseline	1.1 (1.0–1.4)	1.2 (1.0–1.7)	0.0 (–0.1, 0.0)	0.0 (–0.1, 0.1)
Follow-up 2	1.1 (1.0–1.4)	1.3 (1.1–1.6)	0.490	0.528
LDL cholesterol (mmol/L)				
Baseline	2.0 (1.6–2.8)	2.0 (1.6–2.4)	0.1 (–0.1, 0.3)	0.1 (–0.2, 0.3)
Follow-up 2	2.3 (1.7–2.7)	2.1 (1.6–2.5)	0.172	0.631
Albumin (g/L)				
Baseline	45 (44–46)	44 (43–46)	0 (–1, 1)	0 (–1, 1)
Follow-up 2	44 (42–45)	43 (42–44)	0.634	0.696
Total protein (g/L)				
Baseline	71.7 (69.6–74.6)	71.0 (67.8–72.4)	0.7 (–0.9, 2.2)	0.3 (–1.5, 2.0)
Follow-up 2	70.6 (68.8–73.7)	69.4 (67.4–71.1)	0.390	0.759
HbA1c (mmol/mol)				
Baseline	57 (51–65)	52 (47–59)	2 (–2, 5)	1 (–3, 4)
Follow-up 2	60 (52–65)	54 (49–64)	0.353	0.659
Fasting glucose (mmol/L)				
Baseline	9.0 (7.9–10.2)	8.2 (7.0–10.0)	0.2 (–0.8, 1.1)	–0.4 (−1.4, 0.7)
Follow-up 2	9.2 (8.1–10.3)	8.0 (7.3–9.3)	0.739	0.503
Magnesium excretion 24-h urine (mmol/24 h)				
Baseline	3.30 (2.06–4.71)	4.31 (3.09–5.54)	1.88 (1.21, 2.54)	1.96 (1.19, 2.73)
Follow-up 1	4.80 (3.87–7.04)	4.22 (3.06–5.57)	0.003	<0.001
Follow-up 2	4.76 (3.40–6.98)	4.27 (3.15–5.24)		

All analyses were adjusted for the baseline value. The analyses were conducted without and with adjustment for variables that had clinically relevant differences across treatment arms at baseline. For all biochemical parameters measured in blood except T_50_, data were available for 72 and 68 individuals at baseline and follow-up 2, respectively. For magnesium in 24-h urine, this was 73 and 69, respectively. For the serum magnesium analysis, the values at the screening visit were not taken into account.

Abbreviations: CI, confidence interval; cfPWV, carotid-femoral pulse wave velocity; eGFR, estimated glomerular filtration rate; HbA1c, glycated hemoglobin; T_50_, calciprotein crystallization.

1 After log transformation and back-transformation.

The serum magnesium concentration increased over time in the magnesium group: baseline 0.74 (0.71–0.77) mmol/L, follow-up 1 0.77 (0.74–0.81), and follow-up 2 0.78 (0.75–0.81) (Figure 2 and Table 2). The concentration remained stable around 0.81 mmol/L in the placebo group. The difference in change was just short of statistical significance (*P* = 0.073).

### The effect of magnesium citrate on T_50_ (intention to treat)

Despite fluctuations, mean T_50_ at follow-up 1 and at follow-up 2 remained stable in the magnesium group (338 ± 51 and 345 ± 48 min, respectively) (Figure 2 and Table 2). Similarly, mean T_50_ in the placebo group was 336 ± 58 min at follow-up 1 and 355 ± 48 at follow-up 2. There was no significant difference in T_50_ between the magnesium citrate and placebo groups over the whole trial period [ẞ = 6 min (–11, 22), *P* = 0.491], over the first follow-up period [ẞ = 12 (–8, 32), *P* = 0.240] and over the second follow-up period [ẞ = –1 (–21, 19), *P* = 0.937]. Adjusting for clinically relevant differences in baseline variables across treatment arms did not affect the overall treatment effect markedly [ẞ = 7 (–12, 25), *P* = 0.482]. Finally, no effect modification by sex (*P* = 0.837) or history of CVD was observed (*P* = 0.863).

### The effect of magnesium citrate on cfPWV (intention to treat)

In the magnesium group, mean cfPWV increased slightly to follow-up 1 (16.5 ± 2.2 m/s) and was 16.2 ± 2.3 at follow-up 2 (Figure 2 and Table 2). Conversely, mean cfPWV remained stable in the placebo group at follow-up 1 and follow-up 2 (15.4 ± 2.3 and 15.3±2.5 m/s, respectively). There was a significant difference in change in cfPWV between the magnesium citrate and placebo groups over the entire trial period [ẞ = 0.8 m/s (0.1, 1.5), *P* = 0.021] and over the first follow-up period [ẞ = 0.9 (0.0, 1.8), *P* = 0.050], but not over the second follow-up period [ẞ = 0.7 (–0.2, 1.6), *P* = 0.147]. The differences were in favor of the placebo. Additionally, adjusting for time-varying SBP did not affect the overall treatment effect markedly [ẞ = 0.7 m/s (0.0, 1.4), *P* = 0.041]. However, adjusting for clinically relevant differences in baseline variables across treatment arms, that is, adjustment for baseline eGFR, serum magnesium level, HbA1c, and total peripheral calcification score, rendered the overall treatment effect as statistically insignificant [ẞ = 0.5 (–0.2, 1.3), *P* = 0.180]. Finally, no effect modification by sex (*P* = 0.649) was observed, but there was effect modification by history of CVD (*P* = 0.067). The overall stratified treatment effect was ẞ = 0.6 m/s [(–0.2, 1.4), *P* = 0.137] for individuals without compared with ẞ = 2.2 m/s [(0.7, 3.6), *P* = 0.005] with history of CVD. This treatment effect was primarily attributable to a decrease in the placebo arm, whereas there was no increase in the magnesium arm. So, the favorable effect of the placebo was stronger in those with previous CVD.

### The effect of magnesium citrate on biochemical markers (intention to treat)

Of all other biochemical markers, potassium increased significantly over time in the magnesium group compared with placebo [ẞ = 0.2 mmol/L (0.0, 0.3), *P* = 0.020] (Table 2). After adjustment for clinically relevant differences across treatment arms, this treatment effect was no longer statistically significant [ẞ = 0.1 mmol/L (0.0, 0.2), *P* = 0.172].

### Per-protocol analysis

Exclusion of noncompliant individuals in the magnesium group (*n* = 4) and placebo group (*n* = 2) did not yield large differences with the results of the intention-to-treat analyses. For T_50_, the overall treatment effect was ẞ = 4 min [(–12, 21), *P* = 0.625]. For cfPWV, the overall treatment effect was ẞ = 0.8 m/s [(0.1, 1.5); *P* = 0.029].

### (Serious) adverse events

During the trial, 4 serious adverse events and 34 adverse events were reported (Table 3). There was no significant difference in the total number of adverse events across treatment arms (*P* = 0.811). The serious adverse events consisted of cerebrovascular accidents (*n* = 2; magnesium and placebo group), leukemia resulting in death (*n* = 1; placebo group), and angina pectoris (*n* = 1; magnesium group). The 34 adverse events consisted mainly of gastrointestinal complaints (*n* = 17). There was no significant difference in the incidence of gastrointestinal complaints across treatment arms (*P* = 0.563).

**TABLE 3:** Reporting of (serious) adverse events during the trial and stratified per treatment arm

(Serious) adverse events	Total (*n* = 74)	Magnesium (*n* = 37)	Placebo (*n* = 37)	*P* value
Total adverse events	38 (51.4%)	14	24	0.811
Serious adverse events				
Cerebrovascular accidents	2 (2.7%)	1	1	
Leukemia (death)	1 (1.4%)	0	1	
Angina pectoris	1 (1.4%)	1	0	
Adverse events				
Gastrointestinal complaints	17 (23.0%)	7	10	0.563
Decline in eGFR	4 (5.4%)	0	4	
Muscle weakness or cramps	4 (5.4%)	2	2	
Dysregulated HbA1c	3 (4.1%)	1	2	
Incident atrial fibrillation	1 (1.4%)	1	0	
High serum creatinine level	1 (1.4%)	0	1	
Low serum protein level	1 (1.4%)	1	0	
Low creatinine clearance	1 (1.4%)	0	1	
High serum urea level	1 (1.4%)	0	1	
High serum triglyceride level	1 (1.4%)	0	1	

Significant differences in incidence were tested with the χ^2^ test.

Abbreviations: eGFR, estimated glomerular filtration rate; HbA1c, glycated hemoglobin.

## Discussion

This trial demonstrated that 6 mo oral 350 mg magnesium citrate supplementation did not improve T_50_ and cfPWV in individuals with T2DM. In fact, cfPWV appeared to increase slightly in the magnesium group and decrease slightly in the placebo group, especially in those with a history of CVD. These effects on cfPWV may however be explained by *1*) baseline differences between both arms that attenuated the observed difference after adjustment and *2*) the combination of two small contradirectional effects, likely due to natural fluctuations, creating a higher contrast and thus a stronger detrimental treatment effect. The treatment increased 24-h urinary magnesium excretion (as a surrogate for compliance), but did not affect other markers of renal function, lipid- and glucose regulation, and serum mineral status. Finally, serum magnesium levels only increased slightly in the magnesium group relative to the placebo group over time, but just short of statistical significance.

This trial is the first to investigate the effect of magnesium on T_50_ and cfPWV in T2DM with MAC. As such, the study results may only be compared with other study populations. The T_50_ finding aligns with a Danish trial with 8 wk 360 mg and 720 mg magnesium hydroxide supplementation in patients with CKD stage 3–4 [37]. Similarly, no significant low-dose treatment effect was observed, whereas T_50_ significantly improved in the high-dose group. Baseline serum magnesium was slightly higher (0.79 mmol/L) and reached 0.85 mmol/L after 8 wk in the high-dose group, whereas in the present study it increased from 0.74 mmol/L to 0.78 mmol/L after 24 wk. This may be attributable to the higher dosage and the lower eGFR in the Danish trial, leading to more magnesium retention. Two years later, the same research group demonstrated that 4 wk 1.0 mmol/L magnesium dialysate administration significantly improved T_50_ in people on hemodialysis compared with 0.5 mmol/L [49]. In short, it appears that only high-dose magnesium may increase T_50_. This has so far been demonstrated in patients with CKD stages 3–5, who typically have lower T_50_ compared with T2DM and the general population [25]. The discrepancy may therefore be attributed to the low dosage, better renal function (only one participant had an eGFR<45 mL/min per 1.73 m^2^), or to the baseline T_50_ not being low enough to evoke a compensatory treatment response.

Arterial stiffness was assessed by Schutten et al. [50] after administering 450 mg magnesium, and this research group observed no significant effect after 24 wk in individuals who are overweight or have obesity, which is consistent with the present finding. Furthermore, there was no effect of 600 mg magnesium chelate supplementation on cfPWV in females with hypertension treated with hydrochlorothiazide after 6 mo [22]. In contrast, a Dutch study showed that 24-wk supplementation of 350 mg magnesium citrate improved cfPWV in individuals with overweight or obesity [23]. This finding may be explained by the relatively healthier study population in the former study. It is conceivable that the participants had a higher compensatory capacity due to their lower age, better glycemic control, and presumably lower extent of arterial calcification. This corroborates results of a Swiss study, which demonstrated that treating people on hemodialysis with 0.75 mmol/L magnesium dialysate significantly decreased cfPWV after 4 wk compared with 0.50 mmol/L [51]. In short, it appears that magnesium supplementation may have inconsistent effects, depending on population characteristics (e.g., renal function, magnesium status, and arterial calcification), supplementation duration, and dosage. Furthermore, discrepancies might also arise due to methodological differences. The results may be extrapolated to populations with eGFR>45 mL/min/1.73 m^2^ and normomagnesemia with low-dose magnesium supplementation.

Arterial calcification and high arterial stiffness are highly prevalent in T2DM and are associated with high CVD-related mortality and all-cause mortality. As such, intervention studies are warranted to tackle these risk factors. On the basis of this study, 350 mg elemental magnesium supplementation is not effective in improving arterial calcification and stiffness in this specific study population. This null finding may be explained by the inability of magnesium supplements to increase the serum concentration in normomagnesemic individuals with preserved renal function, a too short follow-up period, an insufficiently high dose, or by the suggested low compensatory capacity of the study population due to age, poor glycemic control, and advanced peripheral arterial calcification. Nevertheless, these population characteristics emphasize the urgency to explore alternative strategies to curb the already high CVD risk. Suggestions for future research are to explore other investigational products, to increase follow-up time and/or dosage of magnesium, and/or to investigate individuals with T2DM who do not yet have visible MAC and stiff arteries, have hypomagnesemia, or have reduced renal function. The latter two characteristics are of interest as magnesium handling is impaired in patients with hypomagnesemia and/or eGFR <45 mL/min/1.73 m^2^, and these characteristics should be considered as inclusion criteria in future studies.

Strengths of this study include its double-blind, placebo-controlled RCT design, the low loss to follow-up (7%), and high compliance (92% compliant individuals). Moreover, the study population comprised a clinically relevant, high CVD risk group. Limitations include the inability to create a statistically significant contrast in serum magnesium levels between both groups, suggesting urinary excretion of supplemental magnesium, as supported by the significant increase in urinary magnesium excretion in the magnesium group. As such, the possibility of a type II error cannot be ruled out. Second, clinically relevant baseline differences between treatment arms were observed for eGFR, HbA1c, total peripheral arterial calcification score, and serum magnesium, all indicating a relatively unhealthier magnesium arm. However, the main analyses with adjustment for these variables yielded similar results for T_50_ but an attenuated and statistical insignificant effect for cfPWV. Additional adjustment for these variables is an appropriate method of dealing with confounding attributable to baseline differences. Third, no information on medication use and weight during the trial was available. It is conceivable that participants who started antihypertensive treatment or lost significant amounts of weight during the trial showed a decrease in cfPWV due to loss of intravascular fluid. Fourth, the intervention lasted 6 mo, so the long-term effect of magnesium supplementation remains to be elucidated. Fifth, we did not measure magnesium intake via food or water due to logistic reasons. We did look into overall diet quality and intake of magnesium-rich food groups (vegetables, whole grains, legumes, nuts, dairy, fish, red meat, processed meat), yet the intake and overall diet quality did not differ markedly between groups and did not change over time. Finally, T_50_ was used as endpoint of calcification and CT-based arterial calcification parameters such as mass score were not considered, though it is a better indication of CVD risk. However, a significant treatment effect of magnesium on mass score was not expected as it is hypothesized that 6 mo of magnesium supplementation is insufficient to modify arterial calcification quantity, as arteries can take years to calcify. As such, the T_50_ may serve as a better outcome in a 6-mo trial. Considering the limitations, there is a need for future research, especially in this high CVD risk study population.

To conclude, six-mo oral 350 mg magnesium citrate supplementation did not improve calciprotein crystallization nor arterial stiffness in individuals with T2DM, peripheral MAC, and high arterial stiffness. The null result appears to be the consequence of normomagnesemia in combination with a preserved renal function, leading to increased urinary magnesium excretion. A higher dosage and/or longer follow-up period is recommended in this population. Moreover, future studies should evaluate whether six-mo magnesium citrate might be beneficial in hypomagnesemic individuals with T2DM and reduced renal function.

## Author contributions

The authors’ responsibilities were as follows – RM: designed the research, collected data, analyzed data, interpreted data, and drafted the manuscript; JYX, SPN: collected data; AP: contributed to data acquisition and interpretation; MGV, PAdJ, PJME, JWJB: designed the research, contributed to data acquisition and interpretation; PJME: was the medically responsible investigator; and all authors: critically revised the manuscript, read, approved the final manuscript, agreed to be accountable for all aspects of the work.

## Data availability

Data can be available upon reasonable request. Data request can be sent to The Hoorn Studies Steering Committee at Hoornstudy@amsterdamumc.nl. More information regarding the procedures for contact can be found on www.hoornstudies.com.

## Funding

JWJB is supported by a ZonMW NWO-Vidi grant (91718304).

## Declaration of generative AI and AI-assisted technologies in the writing process

The author(s) declare that no generative AI or AI-assisted technologies were used in the writing of this manuscript.

## Conflict of interest

AP is founder, employee and stockholder of Calciscon AG (Biel, Switzerland), which commercializes the T50 test. All other authors have no conflict of interest.

## References

[1] van Dieren S., Beulens J.W., van der Schouw Y.T., Grobbee D.E., Neal B. (2010). The global burden of diabetes and its complications: an emerging pandemic. Eur. J. Cardiovasc. Prev. Rehabil..

[2] Kramer C.K., Zinman B., Gross J.L., Canani L.H., Rodrigues T.C., Azevedo M.J. (2013). Coronary artery calcium score prediction of all cause mortality and cardiovascular events in people with type 2 diabetes: systematic review and meta-analysis. BMJ.

[3] Rennenberg R.J., Kessels A.G., Schurgers L.J., van Engelshoven J.M., de Leeuw P.W., Kroon A.A. (2009). Vascular calcifications as a marker of increased cardiovascular risk: a meta-analysis. Vasc. Health Risk Manag..

[4] Lanzer P., Hannan F.M., Lanzer J.D., Janzen J., Raggi P., Furniss D. (2021). Medial arterial calcification: JACC state-of-the-art review. J. Am. Coll. Cardiol..

[5] Phan O., Joki N. (2022). Vascular calcification in diabetic kidney disease. Kidney Dial-Basel.

[6] Meer R., Oughzou I., Hoek A.G., Dal Canto E., Blom M.T., Doesburg T. (2024).

[7] van den Beukel T.C., Wolters F.J., Siebert U., Spiering W., Ikram M.A., Vernooij M.W. (2024). Intracranial arteriosclerosis and the risk of dementia: a population-based cohort study. Alzheimers Dement.

[8] Losurdo F., Ferraresi R., Ucci A., Zanetti A., Clerici G., Zambon A. (2021). Association of infrapopliteal medial arterial calcification with lower-limb amputations in high-risk patients: a systematic review and meta-analysis. Vasc. Med..

[9] Ter Braake A.D., Shanahan C.M., de Baaij J.H.F. (2017). Magnesium counteracts vascular calcification: passive interference or active modulation?. Arterioscler. Thromb. Vasc. Biol..

[10] Peride I., Tiglis M., Neagu T.P., Niculae A., Checherita I.A. (2022). Magnesium-a more important role in CKD-MBD than we thought. Diagnostics (Basel).

[11] Ter Braake A.D., Vervloet M.G., de Baaij J.H.F., Hoenderop J.G.J. (2022). Magnesium to prevent kidney disease-associated vascular calcification: crystal clear?. Nephrol. Dial. Transplant..

[12] Ter Braake A.D., Tinnemans P.T., Shanahan C.M., Hoenderop J.G.J., de Baaij J.H.F. (2018). Magnesium prevents vascular calcification in vitro by inhibition of hydroxyapatite crystal formation. Sci. Rep..

[13] Lagier R., Baud C.A. (2003). Magnesium whitlockite, a calcium phosphate crystal of special interest in pathology. Pathol. Res. Pract..

[14] TenHuisen K.S., Brown P.W. (1997). Effects of magnesium on the formation of calcium-deficient hydroxyapatite from CaHPO4.2H2O and Ca4(PO4)2O. J. Biomed. Mater. Res..

[15] Gelli R., Pucci V., Ridi F., Baglioni P. (2022). A study on biorelevant calciprotein particles: effect of stabilizing agents on the formation and crystallization mechanisms. J. Colloid. Interface Sci..

[16] Ter Braake A.D., Eelderink C., Zeper L.W., Pasch A., Bakker S.J.L., de Borst M.H. (2020). Calciprotein particle inhibition explains magnesium-mediated protection against vascular calcification. Nephrol. Dial. Transplant..

[17] Zaslow S.J., Oliveira-Paula G.H., Chen W. (2024). Magnesium and vascular calcification in chronic kidney disease: current insights. Int. J. Mol. Sci..

[18] Srisuwarn P., Sethakarun S., Nongnuch A., Jongjirasiri S., Sritara C., Klyprayong P. (2022). Dialysate magnesium and coronary artery calcification, bone mineral density, and cramping in maintenance hemodialysis: a quasi-experimental study. Kidney Med.

[19] Sakaguchi Y., Hamano T., Obi Y., Monden C., Oka T., Yamaguchi S. (2019). A randomized trial of magnesium oxide and oral carbon adsorbent for coronary artery calcification in predialysis CKD. J. Am. Soc. Nephrol..

[20] Bressendorff I., Hansen D., Schou M., Kragelund C., Svensson M., Hashemi B. (2023). The effect of magnesium supplementation on vascular calcification in CKD: a randomized clinical trial (MAGiCAL-CKD). J. Am. Soc. Nephrol..

[21] Marques B., Klein M., da Cunha M.R., de Souza Mattos S., de Paula Nogueira L., de Paula T. (2020). Effects of oral magnesium supplementation on vascular function: a systematic review and meta-analysis of randomized controlled trials, High Blood Press Cardiovasc. Prev..

[22] Cunha A.R., D'El-Rei J., Medeiros F., Umbelino B., Oigman W., Touyz R.M. (2017). Oral magnesium supplementation improves endothelial function and attenuates subclinical atherosclerosis in thiazide-treated hypertensive women. J. Hypertens..

[23] Joris P.J., Plat J., Bakker S.J., Mensink R.P. (2016). Long-term magnesium supplementation improves arterial stiffness in overweight and obese adults: results of a randomized, double-blind, placebo-controlled intervention trial. Am. J. Clin. Nutr..

[24] Pasch A., Farese S., Graber S., Wald J., Richtering W., Floege J. (2012). Nanoparticle-based test measures overall propensity for calcification in serum. J. Am. Soc. Nephrol..

[25] Pluquet M., Kamel S., Choukroun G., Liabeuf S., Laville S.M. (2022). Serum calcification propensity represents a good biomarker of vascular calcification: a systematic review. Toxins (Basel).

[26] Eelderink C., Te Velde-Keyzer C.A., Frenay A.S., Vermeulen E.A., Bachtler M., Aghagolzadeh P. (2020). Serum calcification propensity and the risk of cardiovascular and all-cause mortality in the general population: the PREVEND study. Arterioscler. Thromb. Vasc. Biol..

[27] Kantauskaite M., Bolten K., Boschheidgen M., Schmidt C., Kolb T., Eckardt K.U. (2022). Serum calcification propensity and calcification of the abdominal aorta in patients with primary aldosteronism. Front. Cardiovasc. Med..

[28] Bundy J.D., Cai X., Scialla J.J., Dobre M.A., Chen J., Hsu C.Y. (2019). Serum calcification propensity and coronary artery calcification among patients with CKD: the CRIC (chronic renal insufficiency cohort) study. Am. J. Kidney Dis..

[29] van der Vaart A., Eelderink C., van Goor H., Hillebrands J.L., Te Velde-Keyzer C.A., Bakker S.J.L. (2024). Serum T50 predicts cardiovascular mortality in individuals with type 2 diabetes: a prospective cohort study. J. Intern. Med..

[30] Meer R., Romero Prats M.L., Vervloet M.G., van der Schouw Y.T., de Jong P.A., Beulens J.W.J. (2024). The effect of six-month oral vitamin K supplementation on calcification propensity time in individuals with type 2 diabetes mellitus: a post hoc analysis of a randomized, double-blind, placebo-controlled trial. Atherosclerosis.

[31] Wang A.Y., Pasch A., Wong C.K., Chu I.M., Tang T.K., Chu J. (2022). Long-term effects of sevelamer on vascular calcification, arterial stiffness, and calcification propensity in patients receiving peritoneal dialysis: the randomized pilot SERENE (sevelamer on vascular calcification, arterial stiffness) trial. Kidney Med.

[32] Smith E.R., Pan F.F.M., Hewitson T.D., Toussaint N.D., Holt S.G. (2020). Effect of sevelamer on calciprotein particles in hemodialysis patients: the sevelamer versus calcium to reduce fetuin-a-containing calciprotein particles in dialysis (SCaRF) randomized controlled trial. Kidney Int. Rep.

[33] Thiem U., Soellradl I., Robl B., Watorek E., Blum S., Dumfarth A. (2021). The effect of phosphate binder therapy with sucroferric oxyhydroxide on calcification propensity in chronic haemodialysis patients: a randomized, controlled, crossover trial, Clin. Kidney J.

[34] Ketteler M., Wiecek A., Rosenkranz A.R., Pasch A., Rekowski J., Hellmann B. (2021). Efficacy and safety of a novel nicotinamide modified-release formulation in the treatment of refractory hyperphosphatemia in patients receiving hemodialysis-a randomized clinical trial. Kidney Int. Rep.

[35] Mohammad J., Scanni R., Bestmann L., Hulter H.N., Krapf R. (2018). A controlled increase in dietary phosphate elevates BP in healthy human subjects. J. Am. Soc. Nephrol..

[36] Tiong M.K., Cai M.M.X., Toussaint N.D., Tan S.J., Pasch A., Smith E.R. (2022). Effect of nutritional calcium and phosphate loading on calciprotein particle kinetics in adults with normal and impaired kidney function. Sci. Rep..

[37] Bressendorff I., Hansen D., Schou M., Silver B., Pasch A., Bouchelouche P. (2017). Oral magnesium supplementation in chronic kidney disease stages 3 and 4: efficacy, safety, and effect on serum calcification propensity-a prospective randomized double-blinded placebo-controlled clinical trial. Kidney Int. Rep..

[38] van der Heijden A.A., Rauh S.P., Dekker J.M., Beulens J.W., Elders P., t’ Hart L.M. (2017). The hoorn diabetes care system (DCS) cohort. A prospective cohort of persons with type 2 diabetes treated in primary care in the Netherlands. BMJ Open.

[39] Kockelkoren R., Vos A., Van Hecke W., Vink A., Bleys R.L., Verdoorn D. (2017). Computed tomographic distinction of intimal and medial calcification in the intracranial internal carotid artery. PLOS One.

[40] Xu J.Y., Falconnet D., van den Berkmortel T., de la Rosa S., Linder V., de Jonge R. (2025). Verification of the T50 calciprotein crystallization test: bias estimation and interferences. Clin. Chem. Lab. Med..

[41] Xu J., Falconnet D., van den Berkmortel T., de la Rosa S., Linder V., de Jonge R. (2024). Accuracy and interferences in the T50 test: assessing the reliability of a novel diagnostic tool. Nephrol. Dial. Transpl..

[42] Wilkinson I.B., McEniery C.M., Schillaci G., Boutouyrie P., Segers P., Donald A. (2010). ARTERY society guidelines for validation of non-invasive haemodynamic measurement devices: part 1, arterial pulse wave velocity. Artery Res.

[43] Butlin M., Qasem A., Battista F., Bozec E., McEniery C.M., Millet-Amaury E. (2013). Carotid-femoral pulse wave velocity assessment using novel cuff-based techniques: comparison with tonometric measurement. J. Hypertens..

[44] Levey A.S., Stevens L.A., Schmid C.H., Zhang Y.L., Castro A.F., Feldman H.I. (2009). A new equation to estimate glomerular filtration rate. Ann. Intern. Med..

[45] Diagnost-IQ (2025). https://www.diagnost-iq.nl/professionals/referentiewaarden-%20verrichtingenlijst.

[46] Heusschen L., Berendsen A.A., Balvers M.G., Deden L.N., de Vries J.H., Hazebroek E.J. (2022). Relative validity of a short screener to assess diet quality in patients with severe obesity before and after bariatric surgery. Public Health Nutr.

[47] de Boer M.R., Waterlander W.E., Kuijper L.D., Steenhuis I.H., Twisk J.W. (2015). Testing for baseline differences in randomized controlled trials: an unhealthy research behavior that is hard to eradicate. Int. J. Behav. Nutr. Phys. Act..

[48] Twisk J.W.R. (2013).

[49] Bressendorff I., Hansen D., Schou M., Pasch A., Brandi L. (2018). The effect of increasing dialysate magnesium on serum calcification propensity in subjects with end stage kidney disease: a randomized, controlled clinical trial, Clin. J. Am. Soc. Nephrol..

[50] Schutten J.C., Joris P.J., Mensink R.P., Danel R.M., Goorman F., Heiner-Fokkema M.R. (2019). Effects of magnesium citrate, magnesium oxide and magnesium sulfate supplementation on arterial stiffness in healthy overweight individuals: a study protocol for a randomized controlled trial. Trials.

[51] Del Giorno R., Lavorato Hadjeres S., Stefanelli K., Allegra G., Zapparoli C., Predrag L. (2020). Consequences of supraphysiological dialysate magnesium on arterial stiffness, hemodynamic profile, and endothelial function in hemodialysis: a randomized crossover study followed by a non-controlled follow-up phase. Adv. Ther..

